# Body image perception, well-being and creativity in Chinese university students: The necessity for a novel course of medical aesthetics

**DOI:** 10.1371/journal.pone.0330260

**Published:** 2025-08-18

**Authors:** Xiangyu Wang, Tianjing Wang, Leyi Fu, Feng Yun, Fan Qu, Fangfang Wang

**Affiliations:** 1 Zhejiang University, Hangzhou, Zhejiang, China; 2 Women’s Hospital, School of Medicine, Zhejiang University, Hangzhou, Zhejiang, China; 3 Teaching and Research Office of Traditional Chinese Medicine, School of Obstetrics and Gynecology, Zhejiang University, Hangzhou, Zhejiang, China; Longgang Otorhinolaryngology Hospital & Shenzhen Key Laboratory of Otorhinolaryngology, Shenzhen Institute of Otorhinolaryngology, CHINA

## Abstract

**Objective:**

Body image perception significantly impacts university students’ well-being and potentially their creativity. Traditional aesthetic education often neglects direct engagement with body image concerns. To address this gap, we developed an innovative general education course, *Aesthetics in Traditional Chinese Medicine and Western Medicine*, integrating medical aesthetics with multidisciplinary perspectives. This study aimed to optimize the curriculum of our course with evidence-based insights by exploring the relationship among students’ self-perception of physical appearance, well-being, and creative self-efficacy.

**Methods:**

A cross-sectional study was conducted among 328 students at Zhejiang University in December 2024. Participants completed validated scales: Negative Physical Self Scale (NPSS), Objectified Body Consciousness Scale (OBCS), WHO-5 Well-Being Index (WHO-5), and Creative Self-Efficacy scale (CSE). Demographic data and course enrollment intention were also collected.

**Results:**

Significant intercorrelations were observed among NPSS, OBCS, WHO-5, and CSE scores. These associations were further influenced by demographic and academic factors, including sex, age, grade, and major. Specifically, female students exhibited higher levels of self-objectification, while lower-year students reported more negative self-perception of physical appearance. Moreover, Life Sciences & Medicine students demonstrated a greater tendency toward negative self-perception, low psychological well-being level, and decreased creative self-efficacy, compared to peers in other disciplines.

**Conclusion:**

Based on these findings, we propose refining the course with targeted educational interventions, fostering positive body image perception, and addressing the specific needs of identified student groups—particularly medical students—to cope with these challenges.

## Introduction

Body image, defined as an individual’s perception and emotional evaluation of their physical appearance [1], significantly influences well-being—encompasses emotional, psychological, and social dimensions [2]. This relationship is mediated by factors such as reduced self-objectification and greater self-compassion, which buffer individuals against societal pressures and internalized beauty ideals [3]. Self-objectification involves perceiving one’s body as an object to be observed and evaluated based on appearance; this is often manifested through body surveillance, which directly leads to feelings of body shame [4,5]. Individuals with high self-objectification tend to prioritize external evaluations over their intrinsic qualities, resulting in greater body dissatisfaction [4,5]. Previous studies indicate that over 50% of university students experience dissatisfaction with their body image [6], negatively affecting their well-being and potentially leading to disordered eating, depression, and self-harm behaviors [7,8].

Creativity, defined as the generation of novel and meaningful ideas or products [9], is linked to well-being through its role in self-expression and problem-solving. Engagement in creative activities fosters emotional regulation, improves self-efficacy, and promotes flow states, all of which contribute to subjective well-being [10,11]. Although the nexus between body image and creativity remains less explored, theoretically, positive body image may enhance creativity by reducing self-consciousness and freeing cognitive resources for exploratory thinking. For instance, individuals with higher body appreciation may feel less constrained by societal norms, enabling more authentic self-expression [12].

Aesthetic education aims to cultivate individuals’ aesthetic sensibilities and enhance their capacity for beauty perception and artistic creation. It plays a pivotal role in fostering human cognitive processes, creative potential, and aesthetic competencies [13], as well as psychological well-being [14]. University students are at a crucial stage of value development and require positive self-perception along with comprehensive self-evaluation [6]. Consequently, guiding students to understand and assess themselves in a holistic, non-objectifying, and person-centered manner, while encouraging healthy self-improvement, constitutes an essential part of higher education [15]. However, traditional aesthetic education, primarily focused on aesthetic theories and artistic forms, pays limited attention to body image and merely indirectly influences self-perception.

Medical aesthetics, a scientific discipline first proposed by Chinese scholars, focuses on human physical beauty and its assessment through the integration of aesthetic principles and medical perspectives [16]. Unlike medical cosmetology and aesthetic medicine, which aim to enhance aesthetic appearance via medical or surgical interventions, medical aesthetics prioritizes cultivating aesthetic awareness through the application of its principles to human beauty [16]. Although initially implemented in medical education, its interdisciplinary nature and rich humanistic implications render it highly suitable for adaptation as a humanities course for both medical and non-medical students. Therefore, building upon the core content of medical aesthetics, we developed a novel general education course, *Aesthetics in Traditional Chinese Medicine and Western Medicine*, at Zhejiang University in 2024. Conceptualized within a multidisciplinary theoretical framework, this course uniquely integrates perspectives from aesthetics, the arts, psychology, and aesthetic medicine within both traditional Chinese and Western paradigms. Designed with the dual goals of enhancing aesthetic competencies (such as aesthetic sensitivity, artistic literacy, and creative imagination) and fostering positive self-perception, the course features a comprehensive syllabus comprising the following key components:

(1) The principles and historical development of aesthetics: Providing a foundational understanding of beauty, artistic appreciation, and aesthetic experience across diverse cultural contexts.
(2) The concept and value of medical aesthetics: Exploring the application of aesthetic principles to healthcare, body image perception, and wellness practices.
(3) Body aesthetics and its research methodologies: Examining the definition, measurement, and interpretation of human beauty in relation to culture, art, fashion, and health.
(4) An introduction to cosmetic treatments and plastic surgeries: Illustrating the medical perspective while highlighting relevant psychological and ethical considerations.
(5) The interdisciplinary relationship between medicine and art: Emphasizing how artistic insight can contribute to clinical empathy, patient-centered care, and therapeutic creativity.

It is noteworthy that the syllabus integrates traditional Chinese medicine (TCM), which deeply rooted in traditional Chinese philosophy, to complement the Western perspective. TCM’s aesthetic thinking is grounded in philosophical concepts such as harmony between humanity and nature, the balance of yin and yang, and internal-external unity. Despite influences from Western culture, these principles remain central to contemporary China’s hybrid conception of body beauty. Additionally, the course introduces TCM approaches within aesthetic medicine, which offer cost-effective and non-invasive alternatives to surgical interventions.

To understand the factors influencing students’ self-perception, well-being, and creativity, as well as the potential interactions among them, we conducted a cross-sectional study among students at Zhejiang University who had not yet taken this course. This pre-implementation investigation aims to gather baseline data essential for refining the course to effectively achieve its core goal of fostering a healthy attitude toward the body image.

## Methods

### Participants

We conducted a cross-sectional study at Zhejiang University, a leading comprehensive university in China. Participants were anonymously recruited through the university’s online forum and social media platforms (e.g., WeChat) from 02/12/2024–05/12/2024. A total of 329 currently enrolled students of Zhejiang University voluntarily participated in the study. Prior to beginning the questionnaire, participants were provided with a description of the anonymous nature of data collection, the use of data for analysis and academic publication, and their right to withdraw at any time without consequences. Participants under 18 years of age were advised to consult their guardians before proceeding and to participate only with their understanding and support. Participants who chose to continue with the survey completed the online questionnaire administered on the afternoon of 05/12/2024 and no personally identifiable information was recorded to ensure confidentiality and anonymity. Of the initial 329 participants, 328 (163 males and 165 females) provided complete and valid data (one was excluded due to incomplete responses, yielding an attrition rate of 0.3%), which were subsequently categorized into the following major groups: Engineering & Technology, Humanities & Arts, Life Sciences & Medicine (including medicine, dentistry, nursing, pharmaceutics, and veterinary medicine), Social Sciences, Natural Sciences, and Business & Management. This study was approved by Zhejiang University and supervised by the School of Obstetrics and Gynecology, Zhejiang University School of Medicine (Course code: MED0540G). All procedures adhered to the tenets of the Declaration of Helsinki.

### Measures

We used the Negative Physical Self Scale (NPSS) [17] to assess participants’ negative perceptions of their bodies. The scale, initially developed and validated in samples of Chinese adolescents and young adults, includes two dimensions: facial appearance (11 items) and general appearance (5 items). The items were rated on a 5-point Likert scale, where 0 represents “never” and 5 represents “always”. Higher scores indicate greater body dissatisfaction. In this study, the Cronbach’s alpha was 0.895 for both dimensions.

The Objectified Body Consciousness Scale (OBCS) [18] was used to assess the extent to which participants perceive their bodies as objects to be observed and evaluated based on appearance. The scale was translated into Chinese by Jackson and Chen and has been modified and validated in a Chinese sample [19]. It consists of two dimensions: body surveillance (8 items) and body shame (6 items). The items are rated on a 7-point Likert scale, where 1 indicates strong disagreement and 7 indicates strong agreement. Higher scores reflect a greater tendency to view one’s body from an outsider’s perspective. In this study, the Cronbach’s alpha values were 0.809 for body surveillance and 0.783 for body shame.

We employed the World Health Organization Five-Item Well-Being Index (WHO-5) [20] to assess participants’ psychological well-being over the past two weeks. The scale has been validated in many countries and the Chinese version of the WHO-5 was used in this study. It consists of 5 items, each rated on a 6-point Likert scale, where 0 represents “never” and 5 represents “every time.” Higher scores indicate better well-being. The Cronbach’s alpha for the scale was 0.893 in this study.

The Creative Self-Efficacy (CSE) scale from the Short Scale of Creative Self (SSCS) was employed to assess participants’ creative self-efficacy [21]. The scale was translated into Chinese by He et al. and has been validated in the Chinese population [22]. Each item is rated on a 5-point Likert scale, with 1 indicating “absolutely not” and 5 indicating “absolutely yes.” Higher scores reflect greater confidence in one’s creative abilities. The Cronbach’s alpha for the scale was 0.891 in this study.

### Data analysis

Data were analyzed using IBM Statistical Package for Social Sciences (SPSS) version 28.0. Descriptive statistics for variables are presented as mean ± standard deviations (SD) or as percentages, depending on the nature of the variable. Chi-square tests, independent samples *t* tests, and Mann-Whitney *U* tests were used to compare differences between sexes and enrollment intentions. One-way ANOVAs were used to compare differences across major groups. Pearson correlation analysis was conducted to examine the relationships between the employed scales. Univariate and multivariate linear regression analyses were performed to assess variable effects on scale outcomes and check for multicollinearity. A significance level of *P* < 0.05 was set.

## Results

### Participant characteristics with sex and major comparisons

As shown in Table 1, the mean age of the participants was 20.15 ± 1.86 years. 56.40% were lower-grade undergraduates (first- and second-year), 32.32% were upper-grade undergraduates (third-year and above), and 11.28% were graduate students. 58.84% came from urban areas and the rest were from rural and suburban areas. The majority (80.79%) were from the more developed southern region of China. Their annual incomes (including parental support, part-time employment, scholarships, financial aid, etc.) ranged from 12,000–324,000 RMB/year with a median of 30,000 RMB/year, while 63.41% of them were below the median.

**Table 1:** Demographic and psychosocial characteristics by sex.

Category	Total (328)	Sex		*P*
		Male (163)	Female (165)	
**Age**	20.15 ± 1.86	20.00 ± 1.74	20.29 ± 1.97	0.158
**Grade**				**0.040**
Lower-grade undergraduates	185 (56.4%)	102 (62.58%)	83 (50.30%)	
Upper-grade undergraduates	106 (32.32%)	44 (26.99%)	62 (37.58%)	
Graduate students	37 (11.28%)	17 (10.43%)	20 (12.12%)	
**Annual income**				0.128
Low annual income (≤30,000 RMB/year)	208 (63.41%)	110 (67.48%)	98 (59.39%)	
High annual income (>30,000 RMB/year)	120 (36.59%)	53 (32.52%)	67 (40.61%)	
**Region**				0.354
Northern China	63 (19.21%)	28 (17.18%)	35 (21.21%)	
Southern China	265 (80.79%)	135 (82.82%)	130 (78.79%)	
**Major**				**<0.001**
Engineering & Technology	122 (37.2%)	83 (50.92%)	39 (23.64%)	
Humanities & Arts	64 (19.51%)	13 (7.98%)	51 (30.91%)	
Life Sciences & Medicine	55 (16.77%)	25 (15.34%)	30 (18.18%)	
Social Sciences	34 (10.37%)	8 (4.91%)	26 (15.76%)	
Natural Sciences	33 (10.06%)	26 (15.95%)	7 (4.24%)	
Business & Management	20 (6.10%)	8 (4.91%)	12 (7.27%)	
**Interest in courses**	112 (34.15%)	49 (30.06%)	63 (38.18%)	0.121
**NPSS**				
Facial appearance	23.8 ± 7.01	23.33 ± 7.23	24.25 ± 6.79	0.234
General appearance	13.58 ± 4.62	13.42 ± 4.66	13.74 ± 4.58	0.528
Total score	37.37 ± 10.28	36.75 ± 10.40	37.99 ± 10.15	0.273
**OBCS**				
Body surveillance	30.58 ± 8.02	29.28 ± 7.93	31.87 ± 7.93	**0.003**
Body shame	20.88 ± 7.08	20.70 ± 7.25	21.07 ± 6.92	0.639
Total score	51.46 ± 11.97	49.98 ± 11.49	52.93 ± 12.30	**0.025**
**WHO-5**	18.30 ± 4.68	18.66 ± 4.97	17.95 ± 4.36	0.173
**CSE**	21.64 ± 4.57	21.96 ± 4.77	21.32 ± 4.36	0.209

Note: The table presents the demographic and psychosocial characteristics of the participants and their comparisons by sex. Data are shown as mean ± standard deviation for continuous variables and as number (percentage) for categorical variables. Bold values highlighting statistically significant results.

Abbreviation: NPSS, the Negative Physical Self Scale; OBCS, the Objectified Body Consciousness Scale; WHO-5, the World Health Organization Five-Item Well-Being Index; CSE, the Creative Self-Efficacy.

Major distribution differed significantly by sex (*P* < 0.001). Males were predominantly enrolled in Engineering & Technology (50.92%), whereas females were most represented in Humanities & Arts (30.91%). Life Sciences & Medicine exhibited a balanced sex distribution. Overall, 34.15% of participants expressed intent to enroll in the course, with no sex-based difference.

For NPSS, no significant sex differences were observed in facial appearance, general appearance, or total scores. Females had significantly higher OBCS scores than males (49.98 ± 11.49 vs. 52.93 ± 12.29, *P* = 0.025, Cohen’s *d* = −0.249, 95% CI: −0.466, −0.031). On the body surveillance dimension, males scored lower than females (*P* = 0.003, Cohen’s *d* = −0.327, 95% CI: −0.544, −0.109), but no significant sex difference was found on the body shame dimension. Scores on WHO-5 and CSE did not differ by sex.

**Fig 1:**
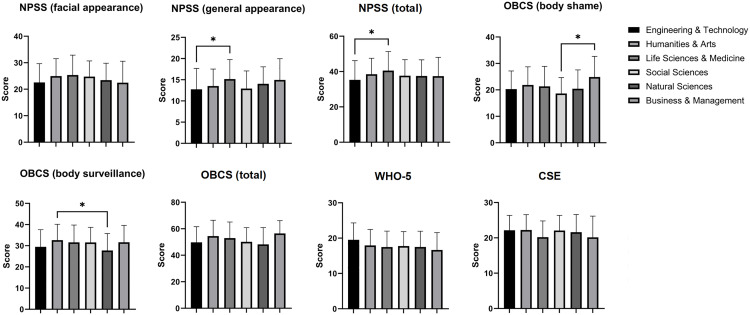
Major comparison of scale scores. Abbreviation: NPSS, the Negative Physical Self Scale; OBCS, the Objectified Body Consciousness Scale; WHO-5, the World Health Organization Five-Item Well-Being Index; CSE, the Creative Self-Efficacy. ***P < 0.001; **P < 0.01; *P < 0.05.

One-way ANOVA indicated significant effects of academic major on psychological measures (Fig 1). While NPSS general appearance scores showed significant variation across majors (*P* = 0.019), neither facial appearance (*P* = 0.082) nor total scores (*P* = 0.054) reached significance. Significant between-major differences were also observed for OBCS (body surveillance: *P* = 0.028; body shame: *P* = 0.032; total: *P* = 0.016) and WHO-5 scores (*P *= 0.012), though no effects emerged for CSE.

### Univariate linear regression analysis

Female students scored significantly higher than males on both total OBCS (*P* < 0.05) and the body surveillance dimension (*P* < 0.01). Younger students scored higher on WHO-5 than older students (*P* < 0.05). Lower-year students had higher WHO-5 scores but lower NPSS (facial appearance and total) scores than higher-year students (all *P* < 0.05). Annual income significantly impacted CSE, with higher income group scoring higher on the scale (*P* < 0.05) (Table 2).

**Table 2:** Univariate linear regression analysis of scales.

Scales Category	NPSS (facial appearance)	NPSS (general appearance)	NPSS (total)	OBCS (body surveillance)	OBCS (body shame)	OBCS (total)	WHO-5	CSE
**Sex**	0.923 (−0.600, 2.446)	0.322 (−0.681, 1.326)	1.245 (−0.987, 3.478)	2.591 (0.868, 4.313)**	0.367 (−1.172, 1.906)	2.958 (0.373, 5.543)*	−0.705 (−1.720, 0.310)	−0.636 (−1.628, 0.356)
**Age**	−0.327 (−0.736, 0.082)	−0.082 (−0.352, 0.188)	−0.409 (−1.008, 0.191)	−0.197 (−0.666, 0.271)	0.208 (−0.205, 0.622)	0.011 (−0.689, 0.711)	0.281 (0.009, 0.553)*	0.061 (−0.206, 0.328)
**Grade**	−1.084 (−2.187, 0.019)	−0.543 (−1.270, 0.185)	−1.627 (−3.243, −0.011)*	0.108 (−1.161, 1.376)	−0.117 (−1.236, 1.002)	−0.009 (−1.902, 1.884)	0.868 (0.134, 1.601)*	−0.307 (−1.029, 0.415)
**Annual income**	−1.071 (−2.651, 0.509)	−0.69 (−1.730, 0.350)	−1.761 (−4.075, 0.553)	0.493 (−1.318, 2.304)	−0.172 (−1.770, 1.426)	0.321 (−2.383, 3.024)	0.694 (−0.360, 1.748)	1.269 (0.245, 2.292)*
**Region**	−0.194 (−2.131, 1.743)	−0.442 (−1.716, 0.831)	−0.636 (−3.474, 2.202)	−0.049 (−2.264, 2.166)	0.426 (−1.527, 2.380)	0.377 (−2.929, 3.683)	0.57 (−0.720, 1.861)	0.199 (−1.063, 1.462)
**Major**								
Humanities & Arts	2.404 (0.290, 4.518)*	0.778 (−0.605, 2.161)	3.182 (0.089, 6.274)*	3.191 (0.784, 5.599)*	1.549 (−0.576, 3.673)	4.74 (1.155, 8.325)*	−1.603 (−3.002, −0.203)*	0.088 (−1.289, 1.464)
Life Sciences & Medicine	2.796 (0.571, 5.021)*	2.426 (0.971, 3.881)**	5.222 (1.968, 8.476)**	2.146 (−0.388, 4.679)	1.014 (−1.222, 3.250)	3.16 (−0.613, 6.932)	−2.052 (−3.525, −0.579)**	−1.949 (−3.398, −0.501)**
Social Sciences	2.186 (−0.470, 4.843)	0.174 (−1.564, 1.912)	2.36 (−1.526, 6.246)	2.082 (−0.943, 5.107)	−1.677 (−4.347, 0.992)	0.405 (−4.100, 4.909)	−1.789 (−3.548, −0.031)*	−0.072 (−1.802, 1.657)
Natural Sciences	0.875 (−1.813, 3.563)	1.293 (−0.466, 3.051)	2.168 (−1.764, 6.099)	−1.691 (−4.751, 1.370)	0.129 (−2.572, 2.830)	−1.562 (−6.119, 2.996)	−2.04 (−3.819, −0.261)*	−0.555 (−2.305, 1.194)
Business & Management	−0.099 (−3.404, 3.205)	2.212 (0.051, 4.374)*	2.113 (−2.721, 6.947)	2.182 (−1.581, 5.945)	4.555 (1.234, 7.875)**	6.737 (1.133, 12.340)*	−2.875 (−5.062, −0.687)*	−1.981 (−4.132, 0.170)

Note: The table presents the results of univariate linear regression analyses for NPSS, OBCS, WHO-5, and CSE scales. The values represent the regression coefficients with 95% confidence intervals. “Engineering & Technology” is used as the reference group for the variable “Major.”

Abbreviation: NPSS, the Negative Physical Self Scale; OBCS, the Objectified Body Consciousness Scale; WHO-5, the World Health Organization Five-Item Well-Being Index; CSE, the Creative Self-Efficacy.

Bold values highlight statistically significant results.

***P < 0.001; **P < 0.01; *P < 0.05.

In terms of major, compared to students from Engineering & Technology, those from other majors scored lower on WHO-5 (all *P* < 0.05). Students from Humanities & Arts scored higher on NPSS (facial appearance and total) and OBCS (body surveillance and total) (all *P* < 0.05). Students from Life Sciences & Medicine scored higher on NPSS (facial appearance, general appearance, and total) (all *P* < 0.05), but scored lower on CSE (*P* < 0.01). Students from Business & Management scored higher on NPSS (general appearance) and OBCS (body shame, and total) (all *P* < 0.05).

### Pearson’s correlation tests

In the Pearson correlation analysis (S1 Table in S1 File), NPSS scores (facial appearance, general appearance, and total) were positively correlated with OBCS scores (body surveillance, body shame, and total) (all *β* > 0, *P* < 0.001). WHO-5 scores were significantly correlated with CSE scores (*β* = 0.539, *P* < 0.001). NPSS (facial appearance, general appearance, and total) and OBCS (body surveillance, body shame, and total) scores were negatively correlated with WHO-5 and CSE scores (all *β* < 0, *P* < 0.05).

### Multivariate linear regression analysis

**Fig 2:**
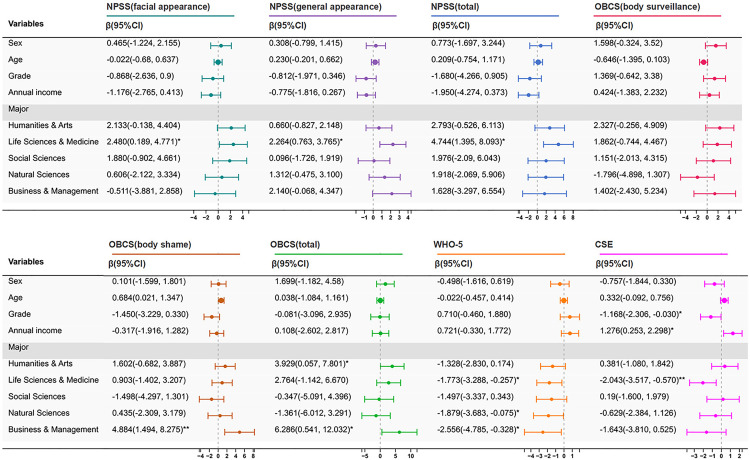
Multivariate linear regression analysis of scales. Note: The figure presents the results of univariate linear regression analyses for the NPSS, OBCS, WHO-5, and CSE scales. The values represent the regression coefficients with 95% confidence intervals. “Engineering & Technology” is used as the reference group for the variable “Major.” Abbreviation: NPSS, the Negative Physical Self Scale; OBCS, the Objectified Body Consciousness Scale; WHO-5, the World Health Organization Five-Item Well-Being Index; CSE, the Creative Self-Efficacy. Bold values highlight statistically significant results. ***P < 0.001; **P < 0.01; *P < 0.05.

Factors identified through univariate linear regression analysis were further analyzed for their effects on each scale using multivariate linear regression (Fig 2). Multicollinearity diagnostics revealed all variance inflation factors (VIFs) ranging from 1.027 to 2.631, indicating acceptable levels of collinearity among predictors in the regression models.

For NPSS (body surveillance, body shame, and total), students majoring in Life Sciences & Medicine scored higher than those in Engineering & Technology (all *P* < 0.05). For OBCS, Business & Management students scored higher on the body shame dimension (*P* < 0.01), while both Humanities & Arts and Business & Management students had higher total scores (both *P* < 0.05).

For WHO-5, students in Life Sciences & Medicine, Natural Sciences, and Business & Management scored significantly lower than those in Engineering & Technology (all *P* < 0.05). For CSE, income showed a positive effect on scores, while grade showed a negative effect (both *P* < 0.05). Moreover, Life Sciences & Medicine students scored lower than Engineering & Technology students (*P* < 0.05).

Further analysis of the effects of NPSS and OBCS on WHO-5 and CSE scores (S2 Table in S1 File) demonstrated that NPSS facial appearance and general appearance dimensions negatively impacted both WHO-5 (both *P* < 0.01) and CSE (both *P* < 0.05). Additionally, grade (*P* = 0.008) and income (*P* = 0.043) remained significant predictors for CSE.

### Course enrollment intention

Significant differences emerged in enrollment intention across majors (*P* = 0.021), with students in Humanities & Arts (50.0%) and Life Sciences & Medicine (38.2%) showing a stronger intention to enroll. The total OBCS (*P* = 0.026) and the body shame dimension scores (*P* = 0.006) were higher in the group with course enrollment intention (Table 3).

**Table 3:** Demographic and psychosocial characteristics by course enrollment intention.

Category	Intended course enrollment (112)	Non-intended course enrollment (216)	*P*
**Sex**			0.121
Male	49 (43.75%)	114 (52.78%)	
Female	63 (56.25%)	102 (47.22%)	
**Age**	20.11 ± 2.04	20.17 ± 1.77	0.784
**Grade**			0.350
Lower-grade undergraduates	67 (59.82%)	118 (54.63)	
Upper-grade undergraduates	34 (30.36%)	72 (33.33%)	
Graduate students	11 (9.82%)	26 (12.04%)	
**Annual income**			0.633
Low annual income (≤30,000 RMB/year)	73 (65.18%)	135 (62.50%)	
High annual income (>30,000 RMB/year)	39 (34.82%)	81 (37.50%)	
**Region**			0.462
Northern China	24 (21.43%)	39 (18.06%)	
Southern China	88 (78.57%)	177 (80.94%)	
**Major**			**0.021**
Engineering & Technology	36 (32.14%)	86 (39.81%)	
Humanities & Arts	32 (28.57%)	32 (14.81%)	
Life Sciences & Medicine	21 (18.75%)	34 (15.74%)	
Social Sciences	12 (10.71%)	22 (10.19%)	
Natural Sciences	6 (5.36%)	27 (12.50%)	
Business & Management	5 (4.46%)	15 (6.94%)	
**NPSS**			
Facial appearance	23.96 ± 7.68	23.71 ± 6.66	0.755
General appearance	13.67 ± 4.48	13.53 ± 4.69	0.799
Total score	37.63 ± 10.71	37.24 ± 10.07	0.743
**OBCS**			
Body surveillance	31.13 ± 7.82	30.29 ± 8.13	0.368
Body shame	22.38 ± 7.28	20.11 ± 6.86	**0.006**
Total score	53.51 ± 11.96	50.40 ± 11.86	**0.026**
**WHO-5**	17.65 ± 4.81	18.64 ± 4.58	0.070
**CSE**	21.47 ± 4.52	21.72 ± 4.60	0.641

Note: The table presents the comparisons of demographic and psychosocial characteristics by course enrollment intention. Data are shown as mean ± standard deviation for continuous variables and as number (percentage) for categorical variables. Bold values highlight statistically significant results.

Abbreviation: NPSS, the Negative Physical Self Scale; OBCS, the Objectified Body Consciousness Scale; WHO-5, the World Health Organization Five-Item Well-Being Index; CSE, the Creative Self-Efficacy.

## Discussion

Medical aesthetics, established as an interdisciplinary course in Chinese medical schools for over two decades, has played an important role in humanity education for medical students [16]. However, its potential within general education has rarely been explored. The course *Aesthetics in Traditional Chinese Medicine and Western Medicine* represents our initial effort to extend this discipline to a broader audience. It employs an interdisciplinary approach to guide both medical and non-medical students in appreciating the practical value of aesthetics and the humanistic dimensions of medicine, while concurrently fostering creative thinking. Crucially, to bridge the gap between traditional aesthetic education and students’ self-perception and well-being, the course promotes a holistic attitude toward the body and health by addressing the following aspects: (1) Avoiding the overvaluation of physical appearance as a primary determinant of personal worth; (2) Appreciating intrinsic values over external features; (3) Raising awareness of the detrimental effects of societal biases related to physical appearance; (4) Cultivating a comprehensive self-evaluation that integrates both internal qualities and external characteristics. Aligned with these objectives, this study investigates the current status of Chinese university students’ self-perception of appearance (assessed via NPSS and OBCS), psychological well-being, and creative self-efficacy, examining their relationships with various demographic factors.

Sex differences were prominent in body consciousness. Female students scored significantly higher than males on OBCS, particularly in the body surveillance dimension. This aligns with prior research confirming women generally experience greater body image concerns [23–25], likely reflecting sociocultural pressures emphasizing female appearance [4,5]. Furthermore, the pervasive influence of social media is recognized to exacerbate self-objectification and amplify negative emotions [26,27]. Consequently, course design and implementation should prioritize addressing the specific needs and experiences of female students.

Lower-year students (first and second years) face a significant transition from high school to university, often encountering challenges in establishing new social lives. They reported significantly higher scores on NPSS, indicating greater dissatisfaction with their physical appearance. Lower-year and younger students also exhibited lower well-being levels, consistent with previous findings [28,29]. However, after controlling for other factors in the multivariate linear regression, the effects of sex, grade, and age on OBCS and NPSS scores were no longer statistically significant. This suggests that the observed associations between these demographic factors and body image perceptions may be mediated or confounded by other variables, calling for further investigation.

A key finding of our study is the significant influence of academic background on students’ self-perception of appearance, a relationship warranting greater attention. Multivariate linear regression analysis revealed that, compared to students in Engineering & Technology, those in both Humanities & Arts and Business & Management reported significantly higher levels of self-objectification. This pattern may be attributed to disciplinary characteristics: Art education often involves training students to view the body as an expressive object, while business contexts frequently place considerable emphasis on personal image and presentation. Interestingly, despite their elevated self-objectification, students from Humanities & Arts did not exhibit significantly lower well-being levels. This resilience might be explained by findings suggesting that fine arts education in higher education can bolster psychological well-being by enhancing creativity and self-efficacy [30].

Conversely, students from Life Sciences & Medicine demonstrated stronger negative self-perception of appearance compared to peers in other majors. It is postulated that their exposure to rigorous standards of physical health within their field may unconsciously heighten body image concerns [31]. Furthermore, this group also reported lower well-being levels. While high academic pressure is an intuitive explanation supported by existing literature [32,33], our analysis suggests that negative body self-perception also plays a significant role. Specifically, NPSS emerged as the only significant predictor associated with WHO-5 in the multivariate linear regression model.

Regarding CSE, students from Life Sciences & Medicine exhibited the lowest scores. While prior research confirms the positive association between creativity and well-being [34], our findings further reveal that CSE correlates significantly with body self-perception and self-objectification. This pattern suggests robust interconnections among body image, well-being and creativity, potentially indicating an integrated theoretical framework linking these constructs. Notably, key mediating factors—such as personality traits, which independently influence both body image and psychological well-being [35,36]—were not examined in this study. Therefore, the observed deficit in medical students’ CSE likely extends beyond our current variables, highlighting the need for comprehensive investigation into the causal mechanisms.

Concerning the course’s potential audience, students with higher self-objectification were more inclined to enroll, with notably higher proportions (over 30%) observed in the Humanities & Arts and Life Sciences & Medicine cohorts. Therefore, although designed as a general education course, its implementation should address the needs of these specific student groups. Focusing on helping them develop healthier body image perceptions offers a pathway to simultaneously enhance their well-being and creative self-efficacy.

Based on our findings, the course should be refined with strategies that reduce body image concerns and cultivate healthier self-perception, as follows: (1) Comparative analysis of aesthetic ideals: Systematically contrast Eastern and Western beauty standards to highlight cultural diversity in aesthetics. This approach encourages critical reflection on internalized societal norms, fostering nuanced self-perception grounded in respect for individual differences. (2) Artistic engagement with anatomy: Integrate medical illustration analysis and creation to reveal the functional beauty of human anatomy. This shifts focus from superficial appearance to appreciation of bodily form and function, promoting body positivity. (3) Mindfulness meditation: Incorporate guided sessions to develop emotional awareness, mitigate appearance-related anxiety, and cultivate inner equilibrium. (4) TCM philosophical wisdom: Emphasize concepts like cosmetic wellness, which links external beauty to internal health, lifestyle harmony, and spiritual cultivation. This reorients focus from external modification to holistic balance. (5) Embodied practice: Teach Baduanjin, a traditional Chinese exercise integrating movement, breath regulation, and mental focus. This enhances bodily awareness, physical well-being, and reinforces body-mind unity.

While the proposed strategies hold promise, several barriers warrant consideration. Faculty capacity presents a primary challenge: instructors require interdisciplinary expertise spanning medical aesthetics, TCM philosophy, and artistic pedagogy, with few academics possessing this integrated skillset. Concurrently, student resistance may arise from discomfort with body image discussions among those with high self-objectification, leading to emotional avoidance. To mitigate these barriers, we recommend faculty training partnerships with medical specialists and art educators, and anonymized reflection protocols to reduce student discomfort.

This study’s strengths include the application of validated scales and diverse participants sampling. However, several limitations have to be acknowledged. First, social desirability bias and response inaccuracy may exist in self-reported questionnaires, potentially compromising measurement validity. Second, participants were recruited exclusively from a single university in Southern China, with online recruitment further limiting representativeness by excluding populations with low digital engagement. Lastly, the cross-sectional approach precludes causal inference. Future research should employ multicenter longitudinal designs to establish temporal relationships and enhance generalizability.

## Conclusion

This study demonstrates significant interrelationships among university students’ self-perception of physical appearance, psychological well-being, and creative self-efficacy, with sex, grade, and majors (particularly Life Sciences & Medicine) emerging as key moderating factors. As medical educators committed to advancing medical humanities, we will carry on with this innovative course to benefit a broader audience of various backgrounds. Based on our findings, appropriate refinements will be implemented to foster positive body image perception and to enhance well-being. For medical students—a population of particular concern—systematically integrated aesthetic curricula represent a promising avenue to improve psychological health and to cultivate creativity.

## Supporting information

S1 File **S1 Table. Pearson correlation analysis between scales.** Abbreviation: NPSS, the Negative Physical Self Scale; OBCS, the Objectified Body Consciousness Scale; WHO-5, the World Health Organization Five-Item Well-Being Index; CSE, the Creative Self-Efficacy. Bold values highlighting statistically significant results. ***P < 0.001; **P < 0.01; *P < 0.05. **S2 Table. Multivariate linear regression analysis of WHO-5 and CSE Scales**. Note: The table presents the results of univariate linear regression analyses for the WHO-5, and CSE scales. The values represent the regression coefficients with 95% confidence intervals. “Engineering & Technology” is used as the reference group for the variable “Major.” Abbreviation: NPSS, the Negative Physical Self Scale; OBCS, the Objectified Body Consciousness Scale; WHO-5, the World Health Organization Five-Item Well-Being Index; CSE, the Creative Self-Efficacy. Bold values highlighting statistically significant results.
(DOCX)

S2 File Row data. (XLSX)
