# Supplementary material for: Body image perception, well-being and creativity in Chinese university students: The necessity for a novel course of medical aesthetics
Source: PLoS One. 2025 Aug 18;20(8):e0330260. doi: 10.1371/journal.pone.0330260 (PMC12360510; doi:10.1371/journal.pone.0330260)
Supplement: S1 File — S1 Table. Pearson correlation analysis between scales. Abbreviation: NPSS, the Negative Physical Self Scale; OBCS, the Objectified Body Consciousness Scale; WHO-5, the World Health Organization Five-Item Well-Being Index; CSE, the Creative Self-Efficacy. Bold values highlighting statistically significant results. ***P < 0.001; **P < 0.01; *P < 0.05. S2 Table. Multivariate linear regression analysis of WHO-5 and CSE Scales. Note: The table presents the results of univariate linear regression analyses for the WHO-5, and CSE scales. The values represent the regression coefficients with 95% confidence intervals. “Engineering & Technology” is used as the reference group for the variable “Major.” Abbreviation: NPSS, the Negative Physical Self Scale; OBCS, the Objectified Body Consciousness Scale; WHO-5, the World Health Organization Five-Item Well-Being Index; CSE, the Creative Self-Efficacy. Bold values highlighting statistically significant results. (DOCX) [file pone.0330260.s001.docx]

|  | **NPSS(facial appearance)** | **NPSS(general appearance)** | **NPSS(total)** | **OBCS(body surveillance)** | **OBCS(body shame)** | **OBCS(total)** | **WHO-5** | **CSE** |
| --- | --- | --- | --- | --- | --- | --- | --- | --- |
| **NPSS(facial appearance)** | 1 |  |  |  |  |  |  |  |
| **NPSS(general appearance)** | **0.543***** | 1 |  |  |  |  |  |  |
| **NPSS(total)** | **0.926***** | **0.820***** | 1 |  |  |  |  |  |
| **OBCS(body surveillance)** | **0.469***** | **0.380***** | **0.491***** | 1 |  |  |  |  |
| **OBCS(body shame)** | **0.355***** | **0.269***** | **0.363***** | **0.255***** | 1 |  |  |  |
| **OBCS(total)** | **0.524***** | **0.414***** | **0.543***** | **0.821***** | **0.762***** | 1 |  |  |
| **WHO-5** | **-0.435***** | **-0.489***** | **-0.516***** | **-0.312***** | **-0.189***** | **-0.320***** | 1 |  |
| **CSE** | **-0.361***** | **-0.453***** | **-0.450***** | **-0.295***** | **-0.119*** | **-0.268***** | **0.539***** | 1 |

**S1 Table. Pearson Correlation Analysis Between Scales**

Abbreviation: NPSS, the Negative Physical Self Scale; OBCS, the Objectified Body Consciousness Scale; WHO-5, the World Health Organization Five-Item Well-Being Index; CSE, the Creative Self-Efficacy.

Bold values highlighting statistically significant results.

****P* < 0.001; ***P* < 0.01; **P* < 0.05.

**S2 Table. Multivariate linear regression analysis of WHO-5 and CSE Scales**

|  | **WHO-5** | | **CSE** | |
| --- | --- | --- | --- | --- |
|  | β(95%CI) | *P* | β(95%CI) | *P* |
| **Gender** | -0.254(-1.223, 0.715) | 0.606 | -0.52(-1.486, 0.446) | 0.290 |
| **Age** | 0.019(-0.364, 0.402) | 0.924 | 0.344(-0.038, 0.726) | 0.077 |
| **Grade** | 0.396(-0.628, 1.420) | 0.448 | **-1.39(-2.411, -0.369)** | **0.008** |
| **Annual income** | 0.33(-0.584, 1.244) | 0.478 | **0.941(0.030, 1.852)** | **0.043** |
| **Major** |  |  |  |  |
| Humanities and Arts | -0.71(-2.017, 0.596) | 0.285 | 0.895(-0.407, 2.197) | 0.177 |
| Life Sciences and Medicine | -0.6(-1.927, 0.726) | 0.374 | -0.98(-2.302, 0.342) | 0.146 |
| Social Sciences | -1.156(-2.757, 0.444) | 0.156 | 0.529(-1.066, 2.125) | 0.515 |
| Natural Sciences | -1.454(-3.025, 0.116) | 0.069 | -0.263(-1.828, 1.303) | 0.742 |
| Business and Management | -1.85(-3.819, 0.119) | 0.065 | -1.083(-3.045, 0.880) | 0.279 |
| **NPSS** |  |  |  |  |
| Facial appearance | **-0.133(-0.215, -0.051)** | **0.002** | **-0.102(-0.183, -0.020)** | **0.015** |
| General appearance | **-0.331(-0.448, -0.215)** | **＜0.001** | **-0.323(-0.439, -0.207)** | **＜0.001** |
| **OBCS** |  |  |  |  |
| Body surveillance | -0.05(-0.114, 0.014) | 0.124 | -0.059(-0.123, 0.005) | 0.072 |
| Body shame | 0.001(-0.067, 0.069) | 0.974 | 0.033(-0.035, 0.101) | 0.336 |

Note: The table presents the results of univariate linear regression analyses for the WHO-5, and CSE scales. The values represent the regression coefficients with 95% confidence intervals. "Engineering and Technology" is used as the reference group for the variable "Major."

Abbreviation: NPSS, the Negative Physical Self Scale; OBCS, the Objectified Body Consciousness Scale; WHO-5, the World Health Organization Five-Item Well-Being Index; CSE, the Creative Self-Efficacy.

Bold values highlighting statistically significant results.
